# Which Algorithm Best Propagates the Meyer–Miller–Stock–Thoss Mapping Hamiltonian for Non-Adiabatic Dynamics?

**DOI:** 10.1021/acs.jctc.3c00709

**Published:** 2023-09-13

**Authors:** Lauren E. Cook, Johan E. Runeson, Jeremy O. Richardson, Timothy J. H. Hele

**Affiliations:** † Department of Chemistry, University College London, Christopher Ingold Building, London WC1H 0AJ, U.K.; ‡ Department of Chemistry and Applied Biosciences, ETH Zürich, Zürich 8093, Switzerland

## Abstract

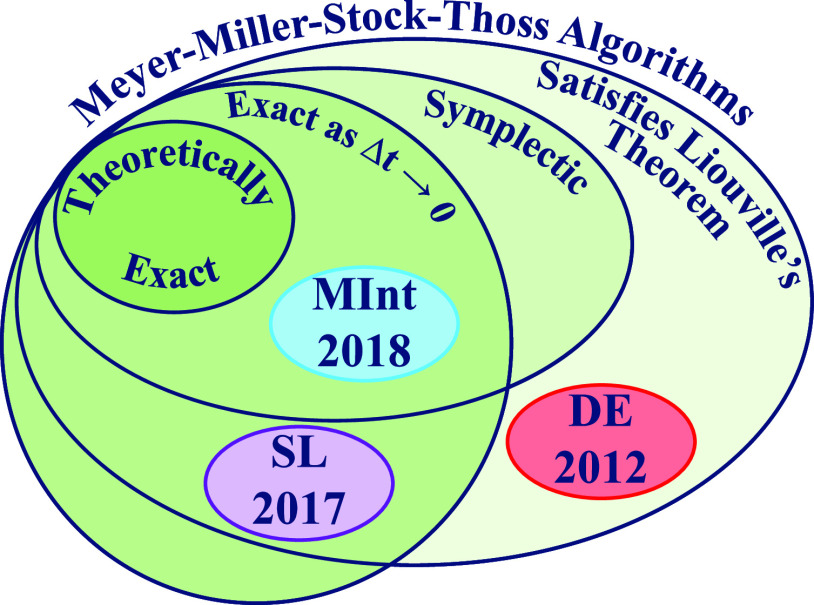

A common strategy to simulate mixed quantum-classical dynamics is by propagating classical trajectories with mapping variables, often using the Meyer–Miller–Stock–Thoss (MMST) Hamiltonian or the related spin-mapping approach. When mapping the quantum subsystem, the coupled dynamics reduce to a set of equations of motion to integrate. Several numerical algorithms have been proposed, but a thorough performance comparison appears to be lacking. Here, we compare three time-propagation algorithms for the MMST Hamiltonian: the Momentum Integral (MInt) (*J. Chem. Phys.*, **2018**, *148*, 102326), the Split-Liouvillian (SL) (*Chem. Phys*., **2017**, *482*, 124–134), and the algorithm in *J. Chem. Phys.*, **2012**, *136*, 084101 that we refer to as the Degenerate Eigenvalue (DE) algorithm due to the approximation required during derivation. We analyze the accuracy of individual trajectories, correlation functions, energy conservation, symplecticity, Liouville’s theorem, and the computational cost. We find that the MInt algorithm is the only rigorously symplectic algorithm. However, comparable accuracy at a lower computational cost can be obtained with the SL algorithm. The approximation implicitly made within the DE algorithm conserves energy poorly, even for small timesteps, and thus leads to slightly different results. These results should guide future mapping-variable simulations.

## 1 Introduction

Theoretical methods for simulating nonadiabatic dynamics are crucial for understanding charge and energy transfer in materials such as organic light-emitting diodes, solar cells, and photosynthetic systems.^1−7^ Experimental spectroscopic techniques have successfully been developed to probe nonadiabatic processes within photovoltaic materials.^8−10^ To analyze the complex dynamics observed, accurate and efficient numerical models are of great importance.^3,9^

Since the 1930s, when Landau and Zener first investigated nonadiabatic dynamics,^11,12^ accurate calculation of nonadiabatic dynamics has been a challenge due to the large computational expense associated with full-quantum solutions.^13−15^ This led to the development of approximate classical-like dynamical frameworks, motivated by the classical linear scaling with degrees of freedom (DoF) compared to the exponential scaling of quantum methods.^16^ Unlike most transformations, discrete quantum DoF do not have an obvious classical counterpart.^16^ Consequently, approximate methods have been developed to recast the quantum system to look classical whilst retaining some quantum properties, often requiring a compromise between accuracy and cost for large systems.

Many methods exist to incorporate discrete quantum DoF into classical frameworks including Ehrenfest dynamics,^17^ approximations to the quantum-classical Liouville equation,^18−20^ the symmetrical quasi-classical windowing method,^21^ surface hopping,^22,23^ and mapping methods such as methods inspired by the Meyer–Miller–Stock–Thoss (MMST) mapping^24−28^ and spin-mapping,^29,30^ including the mapping approach to surface hopping (MASH).^31^ The simplest nonadiabatic method, proposed by Mott (1931), evaluates the quantum electronic dynamics along the classical path of the nuclei, known as the “classical path” approach.^16,18,19,32^ However, this does not include the “back reaction” on the nuclei.^16^ Surface hopping models, originally developed by Tully and Preston (1971), propagate along one adiabatic surface before “hopping” to another.^16,22,23,33,34^ “Hopping” is possible at any point along a trajectory, not just where surfaces cross, destroying the state coherence.^23,26^

In this article, we focus on mapping approaches utilized in various semiclassical methods, where the discrete quantum DoF are mapped onto continuous classical DoF propagated by classical mechanics. The Meyer–Miller mapping developed in 1979,^25^ and later put on a rigorous footing by Stock and Thoss in 1997,^26^ constructs a set of classical variables for the discrete electronic DoF and propagates them with the nuclear DoF using an effective Hamiltonian, the MMST Hamiltonian.^25,26,35^ The electronic dynamics are consistent with the time-dependent Schrödinger equation, and the force exerted on the nuclei is given by the instantaneous values of the electronic variables.^25,35^ The mapping introduces electronic position and momenta, sometimes expressed as action–angle variables, to describe the nuclear motion on coupled potential energy surfaces.^16,26^ This approach maps the time-dependent Schrödinger equation for an *N*-level system to a classical analogue of *N*-coupled harmonic oscillators following Hamilton’s equations of motion.^16,26^

The recently derived spin-mapping approach employs a different mapping formalism but gives a Hamiltonian almost identical to that of the MMST methods.^29,30,36^ The MMST Hamiltonian algorithms compared in this work are thus also applicable to spin-mapping methods. Compared to MMST-based methods, spin-mapping uses a different value of the so-called zero-point energy (ZPE) parameter, introduced by Stock and Müller as a fitting parameter to mitigate ZPE-leakage.^37,38^ Spin-mapping leads to a ZPE as a function of the number of states, with values close to what was previously found optimal when tuned as a free parameter. A partially linearized spin-mapping method has been found to improve accuracy compared to the fully linearized mapping,^39,40^ in particular for spectroscopy.^41^

One application of the MMST Hamiltonian is to calculate dynamical properties at thermal equilibrium by approximating equilibrium time-correlation functions
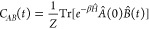
1where the partition function *Z* is given as
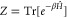
2and the quantum Boltzmann operator at inverse temperature β = 1/*k*_B_*T* is . Semiclassical methods can be used to calculate quantum time-correlation functions, often by utilizing an “Initial-Value Representation” (IVR), resulting in a phase-space integral.^1,26,42^ The semiclassical phase-factor makes correlation function convergence challenging.^1,27,43^ Various versions have been developed to attempt to overcome this, including linearized semiclassical (LSC)-IVR^1,27,44−47^ and Mixed Quantum-Classical (MQC)-IVR.^28,47−51^ However, semiclassical methods often fail at describing nuclear quantum effects.^52^

An alternative method that can capture nuclear quantum effects is to use a ring of multiple classical system replicas (beads) attached by harmonic springs, known as a ring polymer.^53−56^ While the path-integral representation leads to an exact method for static equilibrium properties, the dynamics of the ring polymer can yield short-time approximations to quantum real-time dynamics through methods such as ring-polymer molecular dynamics (RPMD),^53,57−59^ centroid molecular dynamics (CMD),^60−63^ and thermostatted (T)-RPMD.^64−66^ Specifically, RPMD provides, for a single (adiabatic) potential, an approximation to Kubo-transformed time correlation functions while preserving the Boltzmann distribution and is exact in the short-time and classical limits.^58,59,67^ The Kubo-transformed correlation functions allow short-time quantum effects to be included, although the long-time quantum coherence effects are neglected. RPMD can be derived from exact quantum dynamics through a series of approximations *via* Matsubara dynamics,^59,68^ and RPMD transition-state theory is equivalent to true quantum transition-state theory.^67,69−71^ Many extensions have been suggested for multiple states, including mean-field RPMD,^72^ nonadiabatic RPMD (NRPMD), and mapping-variable RPMD (MVRPMD).^3,53,54,73−77^ As NRPMD utilizes the Meyer–Miller Hamiltonian, the algorithms discussed in this work are directly applicable. However, none of these methods alone fulfils the three important criteria of: replicating Rabi oscillations, preserving the quantum Boltzmann distribution, and reducing to classical dynamics in the adiabatic limit.^13,29,78^ While a recently developed ellipsoid spin-mapping fulfils all these limits, its mean-field dynamics was found to be often less accurate for short times than the original spin-mapping.^78^ Further work is still required to find an accurate trajectory-based approach to replicate the true quantum dynamics.

Although mapping Hamiltonians have become increasingly popular for simulating nonadiabatic dynamics, numerical integration of the equations of motion is not straightforward due to coupling between the electronic momenta and nuclear positions. Over the years, various algorithms have been suggested to solve this problem,^1−3^ but as far as we are aware, there has been no published computational comparison of MMST algorithms to determine their properties and accuracy. To address this, in this paper, we compare the symplectic Momentum Integral (MInt) algorithm by Church *et al.* (2018), the Split-Liouvillian (SL) algorithm by Richardson *et al.* (2017), and the algorithm outlined by Kelly *et al.* (2012), referred to here as the Degenerate Eigenvalue (DE) algorithm.^1−3^ Other algorithms exist for this problem, such as Runge–Kutta or the Adams–Bashforth Predictor–Corrector algorithm, which are known to be non-symplectic. We note that even though velocity-Verlet is widely considered to be symplectic for classical systems, directly utilizing velocity-Verlet for the MMST Hamiltonian may not result in a symplectic integration scheme. Individual propagation steps of momenta or position are unlikely to correspond to exact sub-Hamiltonian propagation due to the algebraic form of the MMST Hamiltonian and coupling between the nuclear positions and the mapping momenta. To avoid integrating stiff equations of motion, Wang *et al.* suggested an intelligent canonical transformation.^27^ However, even with this transformation, these approaches are not ideal for propagating mapping variables and will still require short timesteps. We will not consider these algorithms further and instead focus on algorithms which attempt to propagate the mapping variables exactly for an arbitrary timestep.

We seek to compare the algorithms for the simplest possible system for which they can all be compared on an equal footing and for which there already exists results in the literature for qualitative comparison. To this end, we compute position and state autocorrelation functions for a two-state linear vibronic potential with the MMST Hamiltonian (corresponding to the single-bead limit of NRPMD) using these three algorithms.^53^ As many of the methods discussed share similar Hamiltonian forms to that of the MMST, including NRPMD, spin-mapping, Ehrenfest, and some surface hopping models, the results are widely applicable and should inform future computational studies using mapping variable methods.

The article is structured as follows. In Section 2, we provide theoretical background for the three algorithms investigated. In Section 3, we investigate the symplecticity, satisfaction of Liouville’s theorem, accuracy of trajectories and correlation functions, computational cost, and energy conservation. We conclude in Section 4.

## 2 Background Theory

Here, we present the algebraic forms of the three algorithms on an equal footing, such that we can compare properties including the accuracy, symplecticity, and if they satisfy Liouville’s theorem.

Note that a brief theoretical analysis of the MInt and SL algorithms, determining the symplecticity in particular, was previously carried out in the study in ref [^1^]. We extend the work in ref [^1^] to a more thorough computational investigation where we compare the algorithmic performance utilizing a model for which there exists results in the literature and include the DE algorithm. As far as we are aware, the symplecticity has not been rigorously determined for the DE algorithm.

### 2.1 Symplectic Integrators

The Hamiltonian for an *N*-level electronic system in the diabatic representation is
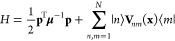
3where the nuclear position and momenta are **x** and **p**, respectively, **V**(**x**) is an *N* × *N* diabatic electronic potential energy matrix in the basis of the electronic states, , and μ is a diagonal matrix of nuclear masses. The classical MMST mapping Hamiltonian in the diabatic representation is^25,26^
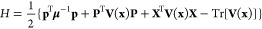
4where **X** and **P** are the electronic position and momenta, respectively. Classical evolution under this Hamiltonian refers to the equations of motion^16,26^
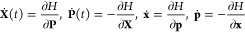
5that constitute a time-dependent Hamiltonian system
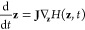
6where , the ∇_**z**_ operator contains the partial derivatives, and **J** is the structure matrix
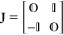
7and  are the identity and zero matrices, respectively.^1,25,79^ A Hamiltonian integrator is said to be symplectic if it fulfils the condition^1,79,80^
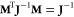
8where **M** is the monodromy matrix. The monodromy matrix is a matrix of differentials that expresses how the time-evolved phase-space variables depend on the initial phase-space variables^81^
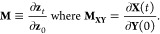
9The monodromy matrix is computed for each timestep and multiplied with the previous timestep matrices to obtain the monodromy matrix for the overall trajectory
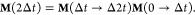
10If the Hamiltonian is split, *H* = *H*_1_ + *H*_2_, then the monodromy matrix is calculated for each timestep according to the algorithmic flow map.^79^ Calculation of the monodromy matrix is not needed for algorithm propagation, only to determine symplecticity and if the theoretical framework requires it. For example, in some SC-IVR methods, the phase-factor can be calculated using elements of the monodromy matrix, where utilizing a symplectic algorithm can improve the stability, aiding convergence and partially mitigating the “sign” problem.^1,82,83^

The symplecticity criterion, eq 8, is a much stricter condition than the conservation of volume phase-space (Liouville’s theorem), which only requires the monodromy matrix determinant to be unity.^1,80,84^ Volume phase-space preservation is a consequence of symplecticity, but this relationship does not necessarily hold the other way around,^79^*i.e.*, volume phase-space preservation is necessary but not sufficient for symplecticity. Instead, symplectic integrators can arise from exact time-propagation of a Hamiltonian system.^79^ Splitting the Hamiltonian into sub-evolutions will also result in a symplectic integrator, provided that each sub-evolution is the exact time-propagation of the relevant sub-Hamiltonian.^1,79^ For example, velocity-Verlet is a classical symplectic algorithm as splitting results in two sub-Hamiltonians that are independent of each other, such that both can be integrated exactly.^80^ The MMST Hamiltonian contains coupling between the electronic momenta and nuclear position, making symplectic time-evolution of the equations of motion challenging and, in general, results in nonlinear dynamics.^1,26^ Symplectic integrators are an advantage for Hamiltonian integration as they have little to no energy drift with time and tend to be more stable at long simulation times.^1^ We also note that the Cayley transform can improve the symplectic stability of algorithms with no additional algorithmic complexity and computational cost, and can be implemented for any path-integral-based scheme.^85^

### 2.2 Algorithmic Overview

The MInt algorithm, by Church *et al.* (2018), was developed to help extend the MQC-IVR method to simulate nonadiabatic dynamics using the MMST mapping.^1^ The name arose as the MInt algorithm exactly solves the **M**omentum **Int**egral with time and as we will show, is the only known symplectic algorithm propagating the MMST Hamiltonian.^1^ The MInt algorithm splits the MMST Hamiltonian into two sub-Hamiltonians, each of which is propagated exactly
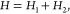
11a
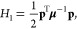
11b
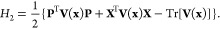
11cThe exact evolution of the sub-Hamiltonians results in symplecticity, and the sub-evolution of *H*_1_ is split into two half-timesteps to improve the time-order error, such that the algorithm is at least a second-order method.^1,79^ Hamilton’s equations of motion are obtained for *H*_1_ and *H*_2_, with the latter being more complicated due to coupling between the nuclear and quantum DoF.^1^ The MInt algorithm can be used on any Hamiltonian containing a sum of Meyer–Miller-like terms and has algebraically been shown to be symplectic, symmetric, second-order in time, and time-reversible.^1^ Fortran code of the MInt algorithm is available in the SC-IVR package on the Ananth Group website.^86^ Recently, the MInt algorithm was utilized by Gardner *et al.* in NQCDynamics.jl, a Julia package for condensed phase nonadiabatic quantum dynamics.^87^ To be able to compare the three algorithms on an equal theoretical footing, we use the D5 form of the MInt algorithm where instead *H*_2_ is split into two half-timesteps^1^
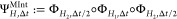
12where Ψ_*H*,Δ*t*_^MInt^ is the approximate flow map comprising exact evolutions of the relevant sub-Hamiltonians, Φ_*H*,Δ*t*_, and propagation is done from right to left. In our notation, Φ refers to exact evolution and Ψ refers to approximate evolution, which may or may not comprise of exact sub-evolutions, consistent with the notation of Leimkuhler and Reich.^79^

The SL algorithm, by Richardson *et al.* (2017), uses the Liouvillian formalism to construct an integrator.^3^ The Liouvillian operator can be generated from the Hamiltonian using a Poisson bracket
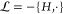
13where the solutions to time-evolution are of exponential form.^84^ We follow the convention in the Matsubara dynamics article^59^ and by Zwanzig^88^ and define the Liouvillian to be real with no factor *i* (the imaginary unit). The MInt algorithm D5 form, eq 12, is equivalent in Liouvillian notation to^1^
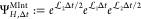
14where evolution requires propagation of the Liouvillians from the right to the left and  are the Liouvillians of *H*_1/2_, respectively. The SL algorithm further symmetrically splits  into electronic (el) and nuclear (**p**) contributions. Hence, the flow map for this algorithm is
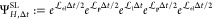
15where
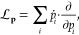
16a
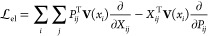
16bare the electronic and nuclear Liouvillians of *H*_2_, respectively, where *i* is the nuclear index and *j* is the electronic index.^1,3^ This uses the approximation
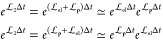
17which is valid in the limit Δ*t* → 0.^3^ Hence, the flow map can also be represented as
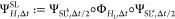
18where  represents the approximate propagation of *H*_2_. Due to symmetric splitting, the two half-timesteps are not equal and are labeled with the superscripts *a* for  and *b* for . For an integrator to be symplectic, it is sufficient but not necessary that the integrator is a sequence of exact sub-Hamiltonian evolutions. Evolution under Liouvillians is not guaranteed to be symplectic but will be if each Liouvillian corresponds to the exact propagation of a Hamiltonian or sub-Hamiltonian.^79^ For an arbitrary timestep, the splitting of *H*_2_ into electronic and nuclear contributions results in holding the electronic position and momenta constant while propagating the nuclear momenta. Hence, the propagation of *H*_2_ is no longer exact or guaranteed to be symplectic.^1^ Church *et al.* (2018) derived the SL monodromy matrix and using the symplecticity criterion, eq 8, confirmed that the SL algorithm is not symplectic.^1^ They stated that there is likely to be an energy drift associated, but this may be small if the adiabatic states are close in energy and weakly coupled.^1^ However, the algebraic proof does not indicate if the SL algorithm will result in unreasonable dynamics such as a very large energy drift when compared to the MInt for a given timestep.

Kelly *et al.* (2012) have also developed an integrator that we refer to as the DE algorithm due to the approximation needed to obtain the final algebraic form. The DE algorithm also splits the Hamiltonian into *H*_1_ and *H*_2_, where the motivation is to enhance stability and minimize the difference between the exact and approximate dynamics.^2^ The algorithm uses MInt-like equations but makes an implicit approximation equivalent to assuming that the diabatic potential matrix eigenvalues are degenerate,^89^ simplifying the calculation of the potential matrix derivative and making the DE algorithm unlikely to be symplectic.^1,2^ The flow map cannot easily be expressed in a Liouvillian form but can be written as
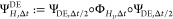
19where Ψ_DE_ is the approximate DE propagation of *H*_2_. The degenerate eigenvalue approximation has previously been utilized in Poisson bracket mapping equation (PBME) simulations of coherent dynamics in photosynthetic systems.^90^ However, the DE approximation is known to cause the state populations to deviate from the exact results for systems with large energy biases.^89^

The three algorithms use equivalent propagation equations for the nuclear position, electronic position and momenta, only differing in the nuclear momenta propagation.^1−3^ The DE algorithm has a similar form to that of the SL algorithm, but the DE algorithm does not hold the electronic position and momenta stationary while propagating the nuclear momenta and has a different diabatic potential matrix differential with respect to the nuclear position.^2,3^ As far as we are aware, the DE algorithm monodromy matrix has not been algebraically determined or tested for symplecticity and Liouville’s theorem. All three algorithms are derived in the diabatic basis; however, as the Meyer–Miller Hamiltonian is known in the adiabatic basis,^91^ it may be possible to re-derive the algorithms in the adiabatic basis.

### 2.3 Propagation of **H**_**1**_

For simplicity, we will consider only one nuclear DoF and two electronic DoF throughout as the generalized multi-dimensional form is known.^1−3^ All three algorithms propagate *H*_1_ in the same way using Hamilton’s equations of motion^1−3^
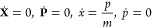
20where *m* is the nuclear mass, such that integration provides the propagation equations
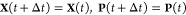
21a
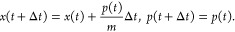
21bWe define our monodromy matrix as
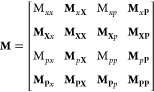
22such that the monodromy matrix for the propagation of *H*_1_ is a triangular matrix of the form
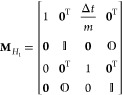
23where **0** = [0,0]^T^ and the determinant is unity, . The propagation of *H*_1_ is symplectic for all three algorithms as .

### 2.4 Propagation of **H**_**2**_

Here, we present and compare the propagation of *H*_2_ for the three algorithms. The diabatic potential matrix is split into a state-independent term, ***U***, and a traceless state-dependent matrix, **Ṽ**, such that **V**(x) = ***U***(x)+**Ṽ**(x). This is as the DE algorithm requires a traceless state-dependent matrix, previously being seen as an advantage as it renders the dynamics invariant to any constant shift of the coupling potential.^2,92^ This gives
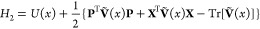
24where  and similarly for **P**. Although the splitting of a traceless state-dependent matrix results in , we have included this term in the derivation for completeness as the MInt and SL algorithms can be used for any choice of splitting, likely resulting in a non-zero trace term. The SL and DE algorithms use approximations to propagate *H*_2_, resulting in different, non-exact nuclear momentum propagation equations.^1−3^

The propagation of *H*_2_ is split into two half-timesteps that sandwich , allowing a fair algorithmic comparison with the original SL form and the equivalent MInt form. We have swapped the order of *H*_1_ and *H*_2_ to test the DE algorithm and in Figures S5 and S6 in the Supporting Information, we show that this has no effect on the symplecticity or energy conservation.

#### 2.4.1 The MInt Algorithm

The MInt algorithm exactly propagates *H*_2_ with time, taking into account the electronic dependence of the nuclear momentum.^1^ Using Hamilton’s equations of motion for half a timestep, following the approach of Church *et al.*, we derive propagation equations
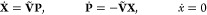
25a
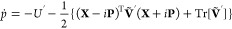
25bwhere the prime denotes the derivative with respect to *x* and the matrix dependence on *x* has been dropped for notational simplicity. To find solutions through integration, the MInt algorithm relies on the fact that  and  are independent of *p* but *ṗ* is dependent on **X** and **P**.^1^ Therefore, solving for  and  and substituting into eq 25b allows  to be found. The electronic position and momenta are found through integration, resulting in
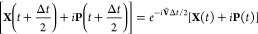
26which can be recast to be entirely real by diagonalizing **Ṽ** into eigenvectors, **S**, and a diagonal eigenvalue matrix, **Λ**, such that .^1^ This results in
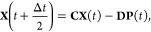
27a
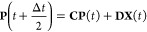
27bfor the propagation of **X** and **P**, where **C** and **D** are^1^
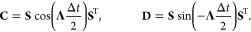
28By defining the derivative of the potential in the adiabatic basis to be  and inserting  identities into the integration of eq 25b, an intermediate equation for the propagation of *p* can be found
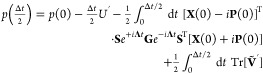
29which can be solved by element-wise integration of
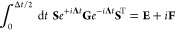
30Defining
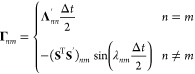
31a
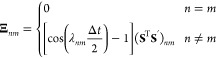
31bwhere λ_*nm*_ = (**Λ**)_*mm*_ – (**Λ**)_*nn*_ , such that
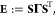
32a
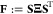
32bwhere **E** is symmetric and **F** is skew-symmetric. The nuclear propagation, where *x* is unchanged, is therefore
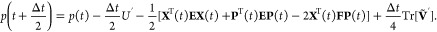
33The extended form for multiple states can be found in Appendix B of ref [^1^]. Church *et al.* have also shown that the MInt algorithm is second-order and exact in the Δ*t* → 0 limit, deviating on . A higher order of accuracy may be obtained through a different splitting of *H*_1_ and *H*_2_.^93^

The MInt monodromy matrix for the propagation of *H*_2_ satisfies Liouville’s theorem and can be found by defining
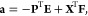
34a
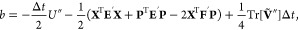
34b
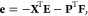
34c
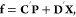
34d
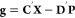
34esuch that the monodromy matrix is^1^
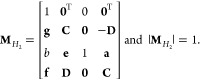
35Propagation under *H*_2_ is symplectic as  satisfies the symplecticity criterion, eq 8, derived by Church *et al.* and shown in Appendix A. As propagation under both *H*_1_ and *H*_2_ is symplectic, the overall propagation for the MInt algorithm is symplectic.^1,79^

#### 2.4.2 The SL Algorithm

The SL algorithm propagates *H*_2_ using the Liouvillian formalism by splitting further into an electronic and nuclear momentum propagation, where eqs 16a and 16b become^1,3^
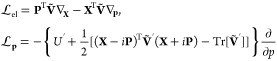
36The electronic propagation resulting from  is equivalent to the MInt algorithm, eqs 27a and 27b, and leaves the nuclear variables unchanged.^1^ results in the following propagation of *p* and leaves all other variables unchanged
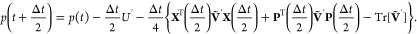
37The difference between the two algorithms arises in the nuclear momentum, where due to the SL symmetric propagation of *H*_2_, the electronic variables are always the half-timestep evolved values for propagation of *p*.^1,3^ This algorithm is exact in the Δ*t* → 0 limit, differing on terms of  as discussed in Appendix B.

The SL monodromy matrices for the propagation of *H*_2_ obey Liouville’s theorem and are
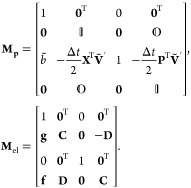
38where^1^
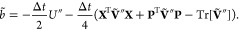
Neither **M**_**p**_ and **M**_el_ satisfy the symplecticity criterion individually, seen in Appendix A.^1^ The combination of **M**_**p**_ and **M**_el_ was shown by Church *et al.* to only be symplectic in the Δ*t* → 0 limit, not for an arbitrary timestep.

#### 2.4.3 The DE Algorithm

The DE algorithm defines **Ṽ** to be traceless, such that the last term of *H*_2_ is ignored.^2^ Here, we will re-frame the algorithm into a MInt-like form using vector notation and outline the degenerate eigenvalue approximation used. We will also investigate the symplecticity and satisfaction of Liouville’s theorem through defining the monodromy matrix, which as far as we are aware has not been done before.

The electronic Hamiltonian equations are the same as above for the MInt and SL algorithms and solved to provide the same overall propagation equations, eqs 27a and 27b. The nuclear momentum propagation equation is
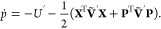
39To solve eq 39, we define an integral, *A*, that requires the degenerate eigenvalue assumption to arrive at the final form by Kelly *et al.*(2)
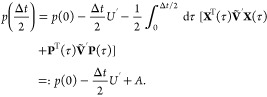
40This can be written in a MInt-like form
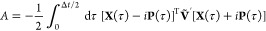
41such that using eq 26
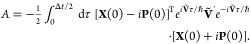
42A transformation is defined into the adiabatic basis using the following overlined variables^2^
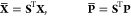
43such that, conversion into the adiabatic basis utilizing the decomposition of **Ṽ** results in
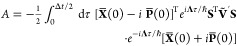
44where  is determined by
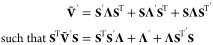
45We can arrive at the form shown by Kelly *et al.* by making the degenerate eigenvalue approximation, which appears to be made between eqs C18 and C19 in ref [^2^]. This approximates that the eigenvalues are equal, . Kim and Rhee determined that this algorithm requires assumption of the degenerate limit agreeing with our DE approximation.^89^ Therefore, the differential of the eigenvalues can be approximated as  such that the derivative of  in the adiabatic basis is obtained through differentiating the identity, 
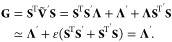
46Inserting this into eq 44 results in
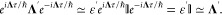
47Hence, the integral below is obtained
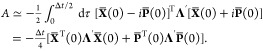
48The final form seen in ref [^2^] has an additional  term when written in matrix form, however, this is just a dummy term. The fact that **Ṽ** is traceless requires that the sum of the eigenvalues is zero, therefore, **Λ** and **Λ**′ are traceless. The overall nuclear propagation in the diabatic basis, having made the DE approximation, is then
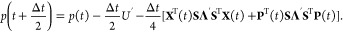
49The DE approximation discards terms to reach eq 49 resulting in the DE algorithm not being exact in the Δ*t* → 0 limit, instead being a zero-order algorithm as derived in Appendix B.

Comparison with the SL and MInt algorithms can determine whether the DE algorithm is likely to be symplectic, where the propagation of *x*, **X** and **P** are equivalent.^1−3^ It can be seen that in the case where the DE approximation holds such that, , the propagation of *p* would be similar to the SL algorithm. However, the DE algorithm uses the initial electronic variables when the SL algorithm uses the half-timestep evolved values. Due to the similarities prior to the DE approximation being made, the DE algorithm is unlikely to be symplectic. To rigorously check the symplecticity, we derive the monodromy matrix by defining
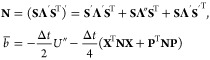
such that
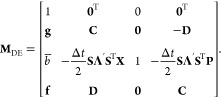
50The determinant of **M**_DE_ can easily be shown to be unity, satisfying Liouville’s theorem. However, the approximate propagation of *H*_2_ is not symplectic as **M**_DE_ does not satisfy the symplecticity criterion, shown in Appendix A. The MInt algorithm is the only algorithm considered here that is both symplectic and satisfies Liouville’s theorem.^1^

In Table 1 below, we present the theoretical results for easy algorithmic comparison. In Appendix C, we prove that all three algorithms conserve electronic probability.

**Table 1:** Summary of the Theoretical Results Obtained Here and Ref [^1^]^a^

Theoretical Results	MInt	SL	DE
*H*_1_ propagation	Exact	Exact	Exact
*H*_2_**X**/**P** propagation	Exact	Exact	Exact
*H*_2_*p* propagation	Exact	Approx.	Approx.
*H*_2_ approximation	None	Split into and	Approx. degenerate eigenvalues of **V**(**x**)
Satisfies Liouville’s theorem	√	√	√
Symplectic	√	×	×
Exact in Δ*t* → 0 limit	√	√	×
Conserves electronic probability	√	√	√

a The *H*_2_ electronic propagation for all algorithms is equivalent. The approximations made in the SL and DE algorithms result in inexact nuclear momentum propagation. However, the SL algorithm is exact in the Δ*t* → 0 limit whereas the DE is not.

## 3 Results and Discussion

The algorithms discussed here have been utilized in the literature but not for the same system to allow direct comparison. We seek to test the algorithms computationally on an equal footing using the same system. Hence, we use a simple two-state linear vibronic potential, also known as a one-dimensional spin-boson model, discussed in this section with the MMST Hamiltonian (corresponding to the single-bead limit of NRPMD) to test the symplecticity, satisfaction of Liouville’s theorem, energy conservation and accuracy of correlation functions. The models defined here also allow qualitative comparison with literature.

### 3.1 Theoretical Models

To compare the approximate dynamics produced by the MInt, SL and DE algorithms, we use the three potential models introduced in ref [^53^]. For these models the potential diabatic matrix is
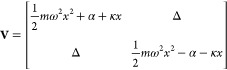
51such that splitting to obtain a traceless **Ṽ** gives
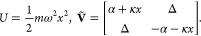
52Reduced units are used where *m* = *ℏ* = ω = 1, so energy is measured in units of the frequency, ω. The models represent bound potentials, defined in Table 2, where Δ is the electronic coupling, 2α is the energy bias between the potential energy surfaces (the asymmetry) and κ is the vibronic coupling, chosen to be 1. Model 1 represents strong electronic coupling, where the nuclear dynamics occur on a longer timescale than the electronic oscillations and nuclear motion occurs in a mean field of the diabatic surfaces. Model 2 has a strong energy bias and the system is in the inverted Marcus regime. Model 3 is a challenging intermediate regime in which the timescales of the electronic and nuclear dynamics are similar.

**Table 2:** Values for the Potential Matrix Constants for Models 1–3 from Ref [^53^]

Model	**α**	**Δ**	Regime
1	0	4	Adiabatic limit
2	2	1	Inverted Marcus regime
3	0	1	Intermediate regime

Models 1–3 were utilized to compute correlation functions using the following distribution, ρ, in the *N*-bead form in refs [^3^ , (53)]. Here, for simplicity, we use the single-bead form
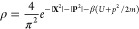
53such that the partition function is
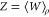
54where *W* = **P**^T^**MXX**^T^**MP** and . Note that *W* is positive definite for the single bead case. Monte Carlo importance sampling is utilized as detailed in Appendix D. Time-independent equilibrium properties are calculated, for example, the population of state *n*(3,53)
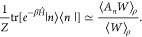
55The derivation is similar to that of eq C4 in Appendix D by summing over all indices except *n*,^3,53^ resulting in
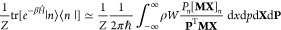
56where
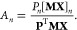
57This approach for obtaining population information is only valid at *t* = 0, whereas the electronic populations can be found at time *t* using^3^
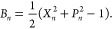
58Correlation functions are calculated through finding an approximation to eq 1, where for the position auto-correlation function  and for the population auto-correlation function  and 
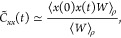
59a
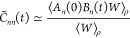
59bwhere *W*(**X**_*i*_(0), **P**_*i*_(0)) and initial conditions are sampled from eq D14 for *J* trajectories. The correlation functions are then averaged over *J* trajectories
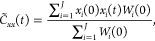
60a
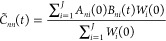
60bwhere index *i* refers to the *i*th trajectory. We can directly compare the three algorithms and qualitatively compare our results with ref [^53^]. We sample the same distribution, with our method outlined in Appendix D, as in ref [^53^]. However, we choose to split the potential matrix such that **Ṽ** is traceless as the DE algorithm requires this. In ref [^53^], the matrix was instead split such that the lowest eigenvalue of **Ṽ** is zero, which in general results in a non-zero trace. To ensure we can compare all three algorithms for the same Hamiltonian, we have used the traceless form leading to small quantitative differences between the results here and in ref [^53^].

### 3.2 Algorithmic Properties

First, we consider the symplecticity and conservation of Liouville’s theorem using Model 1. Church *et al.* determined that the MInt algorithm is symplectic whilst the SL algorithm is not.^1^ In Appendix A, we algebraically show that the DE algorithm is not symplectic. To numerically determine the symplecticity, we define an error matrix, **E**_**r**_, to be
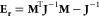
61where for a symplectic integrator the elements of **E**_**r**_, *a*_*ij*_, will all be zero.^94^ The Frobenius Norm is used to track the size of **E**_**r**_
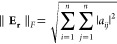
62where the matrix size is *n* × *n*.^94^ To average over many trajectories, we weight by *W*
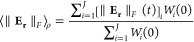
63where *i* refers to the trajectory index. To determine if Liouville’s theorem is satisfied, we evaluate
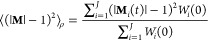
64which will be zero if it is satisfied.^1,80^ By squaring the deviation, we ensure no error cancellation when averaging over trajectories. Satisfaction of Liouville’s theorem is quite a “low bar” for an algorithm, as it is equivalent to the Liouvillian associated with the dynamics being divergenceless.^95^ This can be easily shown for the MInt and SL algorithms but as the DE algorithm cannot easily be expressed in a Liouvillian form, it is still useful to investigate numerically.

**Figure 1:**
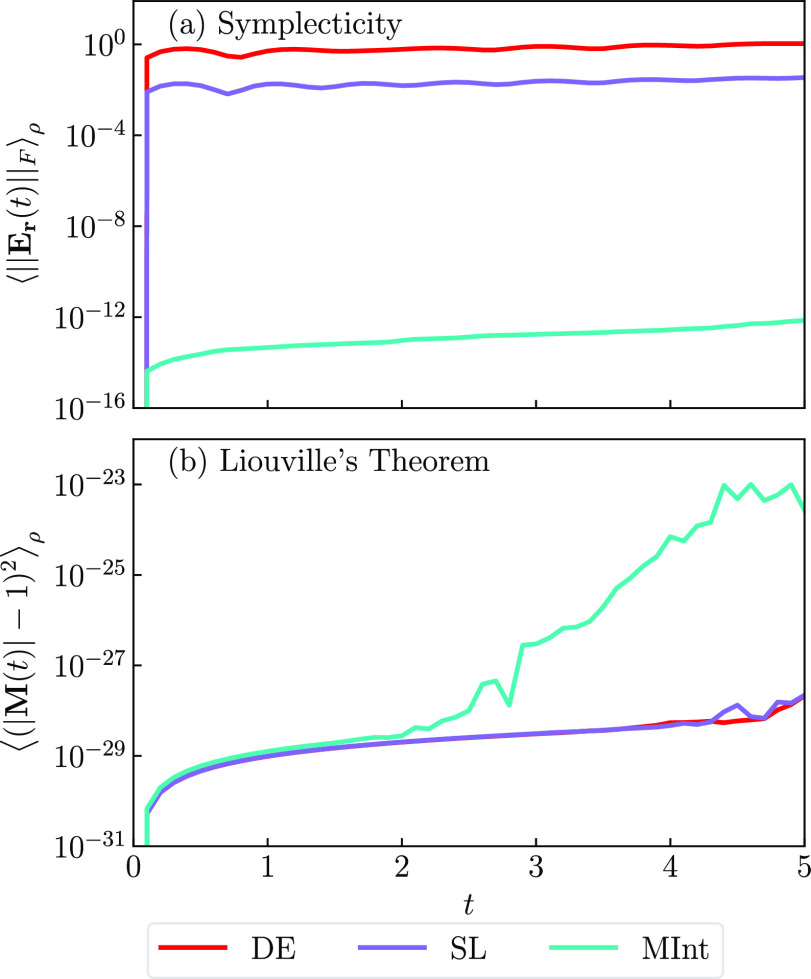
(a) The Frobenius Norm of the symplecticity error matrix and (b) the determinant criterion as a function of time using Model 1 and Δ*t* = 0.1, averaged over a million trajectories using the SL (purple), the MInt (cyan) and the DE (red) algorithms. In (a) the MInt algorithm is seen to be symplectic whereas the DE and SL are not, whereas in (b) all algorithms satisfy Liouville’s theorem. Further detail of (a) with respect to a range of timesteps is presented in Figure A1 in Appendix A.

In Figure 1a, the logarithmic plot of ∥**E**_**r**_∥_*F*_ against time for Model 1 with Δ*t* = 0.1 can be seen. The MInt algorithm (cyan) remains below 10^–12^ for the entire simulation time and is therefore symplectic, with a small build-up of floating-point errors that arise in numerical calculations, in agreement with the literature.^1^ The SL algorithm (purple) increases rapidly to around 10^–2^ and continues to increase indicating that it is not symplectic. The DE algorithm (red) is the least symplectic, being on the order of 1 by the end of the simulation time. In Appendix A, we show the timestep dependence of this error and derive the deviation from symplecticity, using the flow map discussed earlier, to be zero- and second-order for the DE and SL algorithms respectively. Although the SL and DE algorithms are not symplectic, all three algorithms satisfy Liouville’s theorem and conserve volume phase-space, as seen in Figure 1b. We believe that the very slight increase seen for the MInt algorithm is due to additional floating-point error accumulation arising from the more complicated propagation equations.

**Figure 2:**
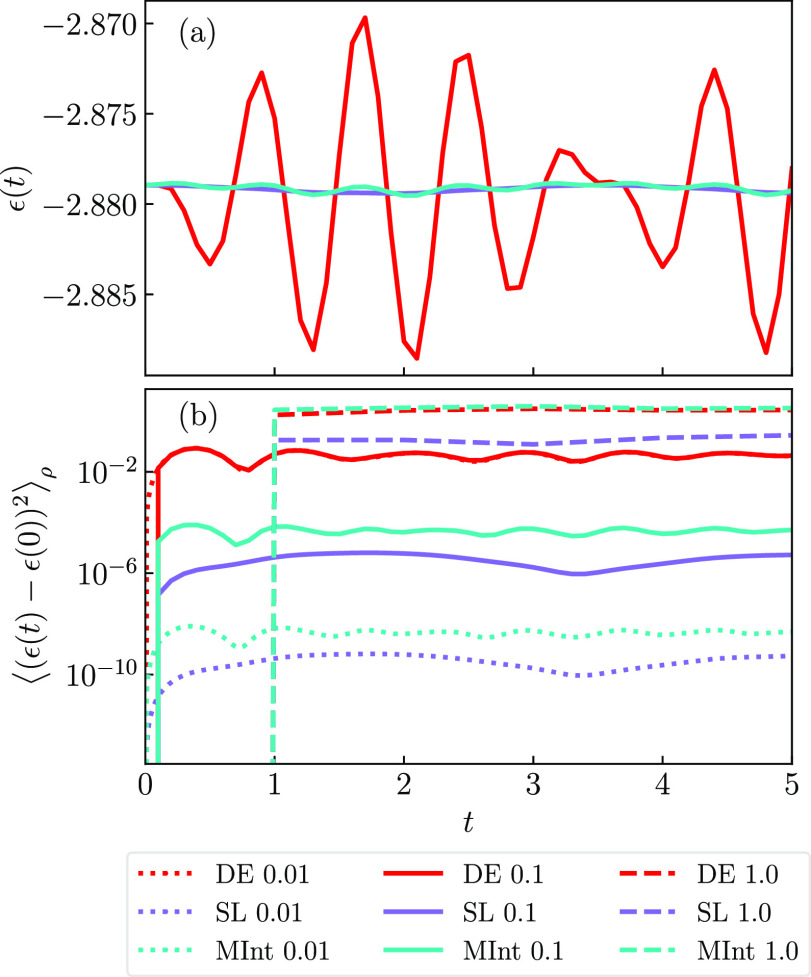
The energy conservation for Model 1 with (a) a single trajectory and Δ*t* = 0.1 (solid) and (b) averaged using Δ*t* = 0.1 (solid), Δ*t* = 0.01 (dotted) and Δ*t* = 1.0 (dashed) with the SL (purple), the MInt (cyan), and the DE (red) algorithms. The DE algorithm has the worst energy conservation and, for this system, the MInt has slightly worse conservation than the SL. The SL and MInt algorithms show improved energy conservation upon decreasing the timestep, unlike the DE.

We now look at energy conservation, Figure 2, where (a) depicts the energy of a single trajectory and (b) averages the energy conservation criterion
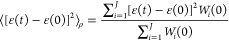
65over trajectories until convergence was observed. We calculate the energy, ε, by evaluating the MMST Hamiltonian, eq 4, at each timestep. Under perfect energy conservation, the criterion should be zero. For a single trajectory, we observe that the DE algorithm has much larger oscillations and does not conserve energy well when compared to the MInt and SL algorithms.

In Figure 2b, we average over many trajectories and consider different timestep sizes. It is seen that the MInt and SL algorithms are second-order as changing the timestep by a factor of 10 increases the criterion by ∼10^4^ as expected.^79^ This agrees with the algebraic determination of the order by Church *et al.*(1) However, the DE algorithm remains the same magnitude for Δ*t* = 0.01 and Δ*t* = 0.1, appearing to not be affected by the smaller timestep. The very coarse Δ*t* = 1.0 is so large that it breaks the trends. The DE algorithm’s poor energy conservation is due to the discarded terms when the DE approximation is made; when these terms are large, the propagation of the nuclear momentum is affected significantly, which then affects all other variables through the propagation equations. The MInt and SL energy conservation is very similar although the SL algorithm is seen to have the smallest energy fluctuations throughout and appears to have a longer period of oscillation for Model 1. This is surprising as one would expect a symplectic algorithm to have better energy conservation compared to a non-symplectic algorithm. However, we note that conservation of energy is not always a good judgement of the algorithm quality. For a symplectic algorithm, the approximate evolution of the exact Hamiltonian is equivalent to the exact evolution of an approximate Hamiltonian that deviates from the exact Hamiltonian on the order of the algorithm.^1,79^ Evolution of a symplectic algorithm results in the energy of the approximate Hamiltonian being exactly conserved, also described as being on the shell of the shadow Hamiltonian. For the MInt, this results in the energy being conserved at exponentially long times with fluctuations on . For Models 2 and 3, we observe that the MInt and SL algorithms have almost identical energy conservation while the DE algorithm is worse, seen in Figures S1 and S2 in the Supporting Information.

### 3.3 Correlation Functions

**Figure 3:**
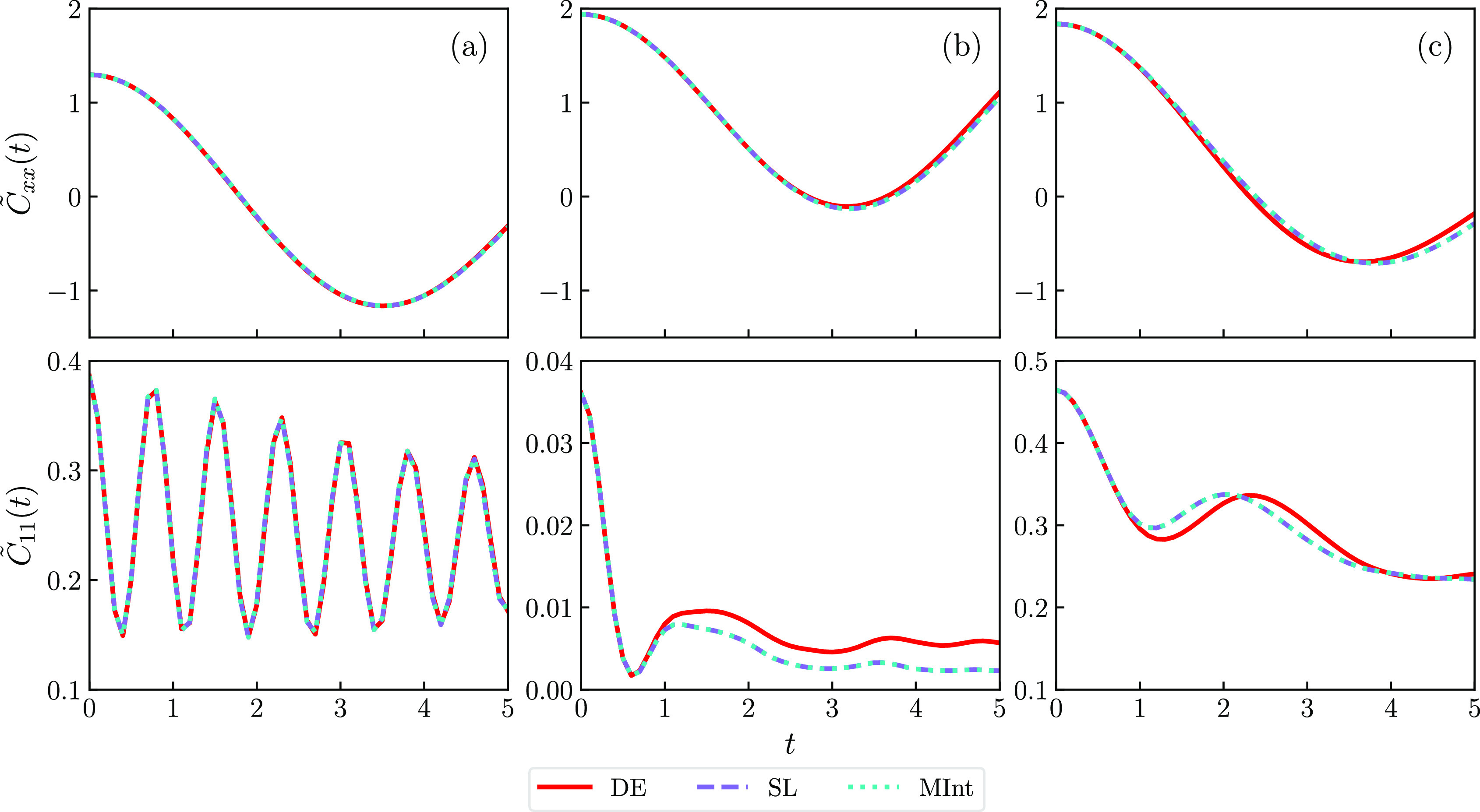
Nuclear position, , and electronic population, , autocorrelation functions for (a) Model 1, (b) Model 2, and (c) Model 3 using Δ*t* = 0.1 with the DE (red solid), SL (purple dashed), and MInt (cyan dotted) algorithms. The SL and MInt give identical results, but the DE algorithm deviates from them in (b, c).

The nuclear position and electronic population autocorrelation functions, eqs 60a and 60b, were calculated for the three models. The fast oscillations of  in Figure 3a indicate that the strong electronic coupling is close to the adiabatic limit and the dynamics tend toward classical evolution on the lower adiabatic surface as *U* is approximately harmonic.^53^ For Models 2 and 3, the weaker electronic coupling reduces the electronic oscillation frequency, producing curves that deviate from the adiabatic result.^53^ In Model 2, the equilibrium population of the first electronic state is quickly lost to the lower energy second state, indicating that the system is almost always on one diabatic surface. The correlation functions obtained using the MInt and SL algorithms qualitatively replicate the dynamics expected from the single-bead calculation in Figure 1 of ref [^53^] for all models. Comparing the three algorithms tested in Figure 3 provides the interesting discovery that the DE algorithm is very accurate for Model 1, despite the lack of energy conservation. This is likely due to averaging with fast electronic oscillations providing the correct convergence. However, for the other models, the DE algorithm predicts the same initial drop in the electronic population autocorrelation functions as the MInt and SL algorithms but starts to deviate from the expected behavior after the minima. The correlation functions have the same shape, indicating a systematic error that likely arises due to the DE approximation. This assumes that the off-diagonal elements of  in the adiabatic basis, **G**, are zero and that the diagonal elements are equal, which is not the case for the SL and MInt algorithms.^1^

While testing energy conservation, we observed that the DE algorithm has more trajectories with poor energy convergence for models with weaker electronic coupling and was particularly poor in the intermediate regime given by Model 3. The MInt and SL algorithms produce the same correlation functions for all the models tested with small timesteps. When testing different timesteps, as seen in Appendix A, both the MInt and SL algorithms are tolerant of a coarse timestep. For Δ*t* = 1.0, we observe that the MInt and SL correlation functions start to differ, with the MInt being closer to the small timestep results. However, the electronic oscillations are not captured well due to aliasing. The MInt and SL algorithms are limited by the model used rather than the algorithmic accuracy.

In Table 3, we provide an overview of the properties tested. The MInt algorithm is the only symplectic algorithm satisfying the symplecticity criterion, providing exact propagation of *H*_1_ and *H*_2_.^1^ The SL and DE algorithms have a non-zero error matrix that increases with time, indicating that neither is symplectic due to the approximations made.^1−3,53^ The lack of symplecticity may result in an energy drift at long simulation times, although this was not observed within the short simulation time tested here.

**Table 3:** Summary of the Computational Results, Which Are Consistent with the Theoretical Results in Table 1

Computational Results	MInt	SL	DE
Satisfies Liouville’s theorem	√	√	√
Symplectic	√	×	×
Energy conservation	good	good	poor
Correlation function accuracy	good	good	poor

The SL algorithm approximates that the electronic variables can be held still while the nuclear momentum is propagated.^3^ The DE algorithm assumes that the eigenvalues of the potential matrix are equal, such that the potential derivative in the adiabatic basis is approximated as .^2^ This results in inaccurate nuclear trajectories that leads to poor energy conservation. However, all three algorithms obey Liouville’s theorem, preserving volume phase-space throughout the trajectories. The MInt and SL have similar energy conservation, where in some cases, the SL has better energy conservation.^1,79^ However, as the MInt is symplectic, it will always fluctuate to the second order, whereas the SL is likely to drift for long simulation times.

The correlation functions produced for the MInt and SL algorithms are very similar to those computed in ref [^53^], with the small differences arising from the different choice of splitting the potential. The DE algorithm converges to a different result for models with weaker coupling, being a good approximation only near the adiabatic limit.

Table S1 in the Supporting Information presents timings of the computational algorithms. One should note that in this case, the bottleneck is the evaluation of the algorithm itself rather than the calculation of the potential matrix. When calculating the monodromy matrix, we observed that the SL algorithm has the lowest computational cost followed closely by the MInt, with the DE algorithm taking the longest time to run. Without calculating the monodromy matrix, the SL is significantly faster, and the MInt and DE take similar times to run.

## 4 Conclusions

In this article, we have tested symplecticity, Liouville’s theorem, energy conservation, and computed correlation functions using the MInt, SL, and DE algorithms for a range of model parameters and timesteps. We find that the computational results agree with our theoretical predictions. If symplecticity is required, for accurate MMST Hamiltonian dynamics with little energy drift, the MInt algorithm should be used. As far as we are aware, the MInt is the only known symplectic algorithm for a general form of the MMST Hamiltonian. However, even though the SL algorithm is not formally symplectic with a finite timestep, it becomes exact in the limit of an infinitesimal timestep. In our tests, it gave comparable accuracy to the MInt algorithm, but at a lower computational cost. We would not recommend the DE algorithm for these models, as it is not exact in the Δ*t* → 0 limit, breaks energy conservation, and introduces errors into the results. This indicates that for the models used here, the degenerate eigenvalue approximation is not valid.

Further work includes integrating the Cayley transform to extend these findings to stable NRPMD simulations.^85^ Additionally, one can apply the MInt algorithm to related dynamical methods such as forward–backward IVR.^51,82^ Further development of the MInt algorithm would be of great interest. For example, as the adiabatic form of the Meyer–Miller Hamiltonian is known,^91^ extension of the MInt algorithm to the adiabatic representation may be possible. Also, one might be able to obtain a higher order of accuracy with the MInt by employing an alternative splitting of *H*_1_ and *H*_2_.^93^

## Figures and Tables

**Figure A1:**
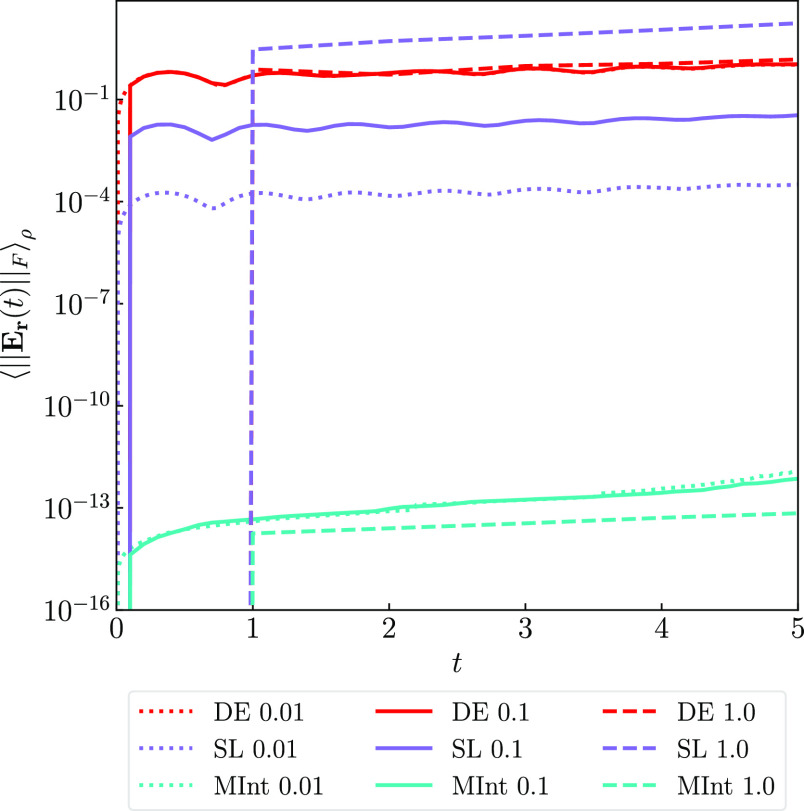
The Frobenius Norm of the error matrix averaged until convergence with the SL (purple), the MInt (cyan), and the DE (red) algorithms using timesteps of Δ*t* = 0.1 (solid), Δ*t* = 0.01 (dotted), and Δ*t* = 1.0 (dashed). The MInt algorithm is seen to be symplectic and does not change with timestep. The SL algorithm is seen to be second-order with respect to time and the DE algorithm is zero-order, in agreement with the theoretical predictions.

## Appendices A

### Symplecticity

The symplecticity criterion can be algebraically determined for the three algorithms, where the MInt and SL have already been derived by Church *et al.* (2018). It is sufficient to say that under the Hamiltonian splitting into *H*_1_ and *H*_2_, the symplecticity criterion becomes^1,79^

A1

For example, following the method by Church *et al.*,^1^ is eq 23 for all three algorithms. This can be derived from the propagation equations, eqs 21a and 21b, where M**_XX_** = M_*xx*_ = M_*pp*_ = M**_PP_** = 1 and

A2

Evaluating the symplecticity criterion results in

A3

such that the propagation under *H*_1_ is symplectic.^1^

The symplecticity for *H*_2_ is derived separately for all three algorithms due to the different nuclear propagation equations, where the MInt and SL algorithms had previously been derived by Church *et al.*(1) For the MInt algorithm,  is eq 35 where the symplecticity criterion becomes

A4

with

where Church *et al.*(1) previously determined that **h** ≡ **0** and **j** ≡ **0** ∀ **X**, **P**.

For the SL algorithm, propagation of *H*_2_ is split further into nuclear and electronic contributions for which the monodromy matrices are eq 38. Evaluating the symplecticity criterion for **M**_**p**_

A6

which will only be symplectic if .^1^ Evaluating for **M**_el_

A7

where eqs A5a and A5b have been used in conjunction with the fact that **h** ≡ **0** and **j** ≡ **0** ∀ **X**, **P**.^1^

Church *et al.* note that **a**, **e** ≠ **0**^T^ so evolution under  and  is not symplectic. The combined evolution under  and  does not provide error cancellation that restores symplecticity. We find that numerically the difference between  and **M**_el_**M**_**p**_ is on , in agreement with the literature.^1^ The combination of **M**_**p**_**M**_el_ also provides the same result. This means that the propagation of **M**_**p**_**M**_el_ will be symplectic in the Δ*t* → 0 limit, but it will not be for an arbitrary timestep.

For the DE algorithm, the monodromy matrix, **M**_DE_, for the approximate propagation of *H*_2_ has been derived for the first time as eq 50. Using eqs 34a–34e with **h** ≡ **0** and **j** ≡ **0** ∀ **X**, **P**, the symplecticity criterion becomes

A8

which will not be symplectic for the same reasoning as for the SL algorithm.

To derive the order of the difference between  and **M**_DE_, following the approach of Church *et al.*,^1^ we define

A9a

A9b

such that, the symplecticity criterion will be met if **a̅** ≡ a and **e̅** ≡ e. Expanding in coefficients of **X** and **P** leads to

A10a

A10b

Rotating to the diabatic basis gives

where Γ and Ξ are given in eqs 31a and 31b. Evaluating element-wise in powers of Δ*t*

Hence, the order of the difference between  and **M**_DE_ is . The SL algorithm, **M**_el_**M**_**p**_, is one order higher than the DE algorithm, **M**_DE_, so the symplecticity is better as seen in Figures 1 and A1.

We find that, numerically, when considering the whole propagation of *H* using the SL algorithm, the initial difference in symplecticity between the MInt and SL algorithms scale on . This is due to some error cancellation arising from the symmetric splitting of *H*_2_. Hence, when propagating for a given length of time, the order is , seen in Figure A1, where changing the timestep by a factor of 10 results in a change in the symplecticity by ∼10^2^. For the DE algorithm, the initial difference in symplecticity with the MInt algorithm scales on . Therefore, for a given length of time, the order is . Figure A1 corroborates this as the DE algorithm appears to be zero-order for all the timesteps tested. The symplecticity does not improve with smaller timesteps.

We note that for symplectic methods, *i.e.*, the MInt algorithm, the **E**_**r**_ matrix will be smaller for coarser timesteps due to fewer computations where floating point errors accumulate. We see this when comparing the Δ*t* = 1.0 and Δ*t* = 0.1 results for the MInt algorithm. This trend is not seen as clearly for Δ*t* = 0.01 and Δ*t* = 0.1 due to the log scale of the plot, but it is present by the end of the simulation time. In contrast, the **E**_**r**_ matrix is typically larger for coarser timesteps for non-symplectic methods as the deviation is an order of Δ*t*, as shown above with the zero- and second-order behaviour of the DE and SL algorithms respectively.

## B

### Exactness in Δ*t* → 0 Limit

The exact Hamiltonian propagation can be expressed as a Liouvillian with the flow map

B1

where using eq 13

B2

The Maclaurin expansion of the exact dynamics is

B3

such that, to obtain the short-time limit, we truncate the series at .

The MInt algorithm has previously been proved by Church *et al.*(1) to deviate from exact dynamics at terms on  and is at least a second-order method. The MInt algorithm is therefore exact in the Δ*t* → 0 limit. As all three algorithms have the same propagation of *x*, **X**, and **P**, the only step that differs for the SL and DE algorithms is the propagation of nuclear momentum. Hence, we focus on the deviation of this propagation from the exact dynamics, where the short-time propagation of *p* for a whole timestep is

B4

B5

B6

If we stop here, this is the SL propagation of *p*, eq 37. The SL algorithm is exact in the short-time limit, deviating by terms of at least  and therefore is at least a first-order method.

To compare with the DE algorithm, we insert eq 45 into the short time limit in the previous equation, and as the DE requires a traceless **Ṽ**, we discard the trace term such that

B7

It can be seen that this is equivalent to the DE algorithm, eq 49, if the following terms are discarded

B8

These terms vanish when the DE approximation is made and are . One might argue that a prior nuclear position propagation, eqs 21a and 21b, may restore these terms. However, this would result in **Λ**′(*x*) being replaced with **Λ**′(*x* + *p*Δ*t*/*m*). Using a Taylor expansion, we can show that

B9

such that, any correctional terms would be at least  due to the factor of Δ*t* in eq B7. This same logic holds for **X** and **P**. Hence, the DE algorithm differs from the exact evolution at , meaning it is zero-order and is not exact in the Δ*t* → 0 limit.

## C

### Conservation of Electronic Probability

To prove that all three algorithms conserve electronic probability, we first define

C1

Taking the value at time *t*

a translation back in time can be achieved with eq 26, resulting in

which is equivalent to eq C2. As all three algorithms propagate eq 26 exactly, this derivation holds and all three algorithms conserve electronic probability.

## D

### Sampling

Following a similar derivation of the partition function as in refs [^3^] and [^53^], the sampling method used in this work is derived. Instead of formulating in the limit of an infinite number of ring-polymer beads, we derive the partition function in the single-bead case. The wavefunctions of the singly excited oscillator (SEO) states are known in the position and momentum bases^3^

D1

D2

where ⟨*n*|**X**⟩ = ⟨**X**|*n*⟩* and likewise for **P**. The partition function, , is expanded as a Trotter product where *N* = 1 for the one-bead case

D3

Inserting the projection operator  and summing over the two electronic levels in our model

D4

where the imaginary-time free-particle propagator for *N* = 1 is^3^

D5

such that

D6

Separating out the state-independent potential, *U*

D7

The sum can be rewritten in matrix notation

D8

where  and using the cyclic properties of the trace

D9

which is a scalar quantity. Using the Gaussian integral identity

D10

such that *p* can be inserted into the integral

D11

resulting in the probability distribution, ρ, eq 53, where *W* is positive-definite in the single-bead case as

D12

therefore

D13

This means that , which will always be positive. We sampled Gaussian distributions for electronic variables where μ = 0 and , and classical distributions for nuclear variables where μ_*x*_ = 0 and , and μ_*p*_ = 0 and , such that the sampled distribution is

D14

To obtain the required distribution, eq 53, the following relationship is established

D15

Hence, we need to correct calculated observables to average over the required distribution. We can thus define the average of an observable over the distribution, , that may be a function of initial position and momenta, i.e.,  as

D16

The integral over the sampled distribution can be replaced with a sum over the number of samples taken, *J*,

The partition function can be calculated as

D19

## Supporting Information Available

Additional energy conservation plots for Models 2 and 3, comparison of correlation functions using different timesteps, and properties of the DE algorithm in the original form (PDF)
