# Supplementary material for: Which Algorithm Best Propagates the Meyer–Miller–Stock–Thoss Mapping Hamiltonian for Non-Adiabatic Dynamics?
Source: J Chem Theory Comput. 2023 Sep 13;19(18):6109–25. doi: 10.1021/acs.jctc.3c00709 (PMC10536990; doi:10.1021/acs.jctc.3c00709)
Supplement: Supplementary file 1 — ct3c00709_si_001.pdf [file ct3c00709_si_001.pdf]

Supporting Information: Which Algorithm Best Propagates the Meyer-Miller-Stock-Thoss Mapping Hamiltonian for Non-Adiabatic Dynamics?

Lauren E. Cook,[†] Johan E. Runeson,[‡,¶] Jeremy O. Richardson,[‡] and Timothy J. H. Hele[*,†]

†*Department of Chemistry, University College London, Christopher Ingold Building, London WC1H 0AJ, United Kingdom*

‡*Department of Chemistry and Applied Biosciences, ETH Zürich, 8093 Zürich, Switzerland*

¶*Current Address: Department of Chemistry, University of Oxford, Physical and Theoretical Chemistry Laboratory, South Parks Road, Oxford OX1 3QZ, United Kingdom*

September 1, 2023

In this Supporting Information, we have included additional energy conservation plots for Models 2 and 3 [1] using the MInt, SL and DE algorithms, [2–4] a comparison of correlation functions using different timesteps and the properties of the DE algorithm in the original propagation form.

Additional Energy Plots

We have included the energy plots for Models 2 and 3 from ref. [1] as Figures S1 and S2 respectively.

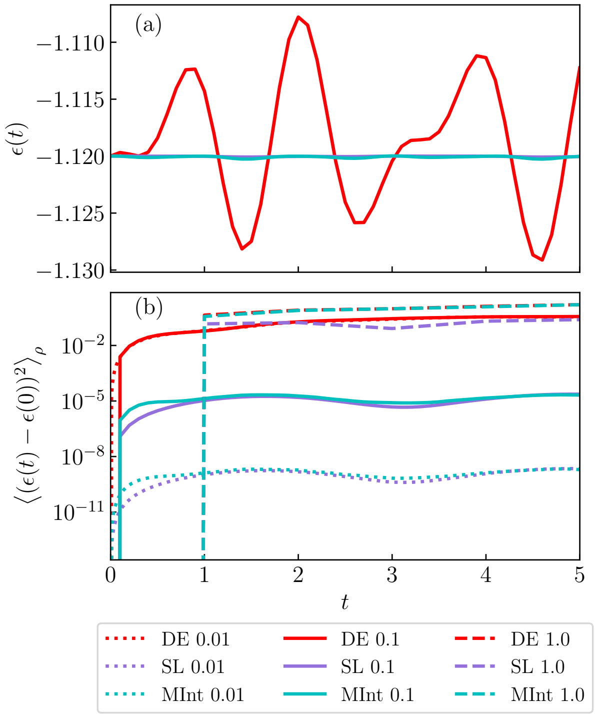

Figure S1: The energy conservation for Model 2 with (a) a single trajectory where $\Delta t = 0.1$ (solid) and (b) averaged using $\Delta t = 0.1$ (solid), $\Delta t = 0.01$ (dotted) and $\Delta t = 1.0$ (dashed) with the SL (purple), the MInt (cyan), and the DE (red) algorithms. Here, we observe that the MInt and SL algorithms give almost identical energy conservation whilst the DE deviates.

As the electronic coupling decreases between the models, the frequency of the energy oscillation for the DE algorithm for a single trajectory also decreases. The MInt and SL algorithms have very similar energy conservation and are second-order for both models at all tested timesteps. Again, we see that the DE algorithm is zero-order with respect to the propagation time (as seen from the fact that it gives the same error at $\Delta t = 0.1$ and $\Delta t = 0.01$).

The MInt and SL algorithms have very similar energy conservation for the two models shown

here. This is surprising as one would expect a symplectic algorithm to have better energy conservation. For a symplectic algorithm, the dynamics are equivalent to an exactly conserved approximate Hamiltonian that deviates from the exact Hamiltonian on the order of the algorithm. However, while a symplectic algorithm fluctuates in energy, it does not drift. That does not mean that a non-symplectic algorithm will have poor energy conservation, and that is what we see here with the SL algorithm.

Overall, we conclude that the SL and MInt algorithms have good energy conservation, although this deteriorates with coarser timesteps. The DE algorithm has poor energy conservation that is zero-order until it breaks at coarse timesteps.

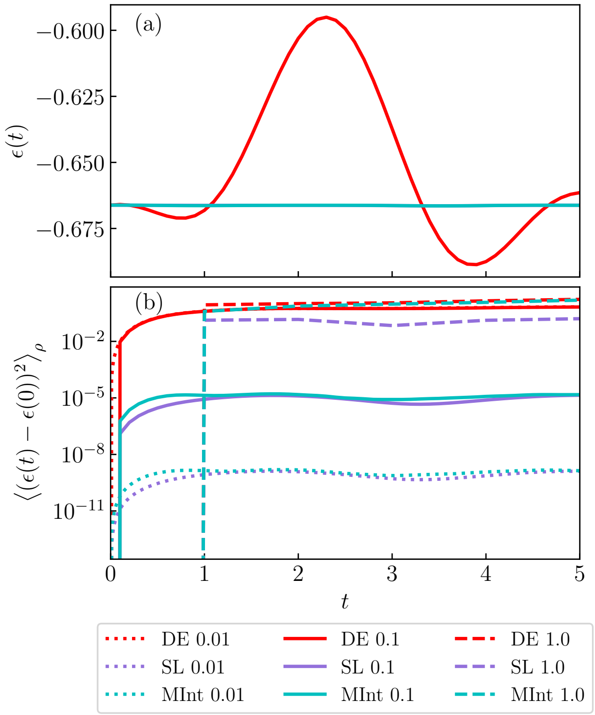

Figure S2: The energy conservation for Model 3 with (a) a single trajectory where $\Delta t = 0.1$ (solid) and (b) averaged using $\Delta t = 0.1$ (solid), $\Delta t = 0.01$ (dotted) and $\Delta t = 1.0$ (dashed) with the SL (purple), the MInt (cyan), and the DE (red) algorithms. Again, the MInt and SL algorithms give almost identical energy conservation whilst the DE deviates.

Additional Timesteps for Correlation Functions

The correlation functions seen in Figures S3 and S4 indicate the accuracy of two different timesteps tested; $\Delta t = 0.01$ and $\Delta t = 1.0$. The results for $\Delta t = 0.1$ are shown in Figure 3 of the main paper. We see that $\Delta t = 0.01$ and $\Delta t = 1.0$ produce very similar results, where the MInt and

SL are identical. When $\Delta t = 1.0$, the nuclear position autocorrelation function is quite accurately captured but the electronic population correlation function suffers from aliasing for Models 1 and 2 as the oscillations are faster than the timestep used. We see that the MInt and SL algorithms no longer produce the same correlation functions when $\Delta t = 1.0$. The MInt algorithm is closer to the results seen for the smaller timesteps and thus is more accurate. This likely arises from the more accurate propagation of H_2. The DE algorithm converges on the same value with the smaller $\Delta t = 0.01$ as for $\Delta t = 1.0$, indicating that this algorithm does not improve with a smaller timestep (it is not exact in the $\Delta t \to 0$ limit) and the approximation leads to a systematic error.

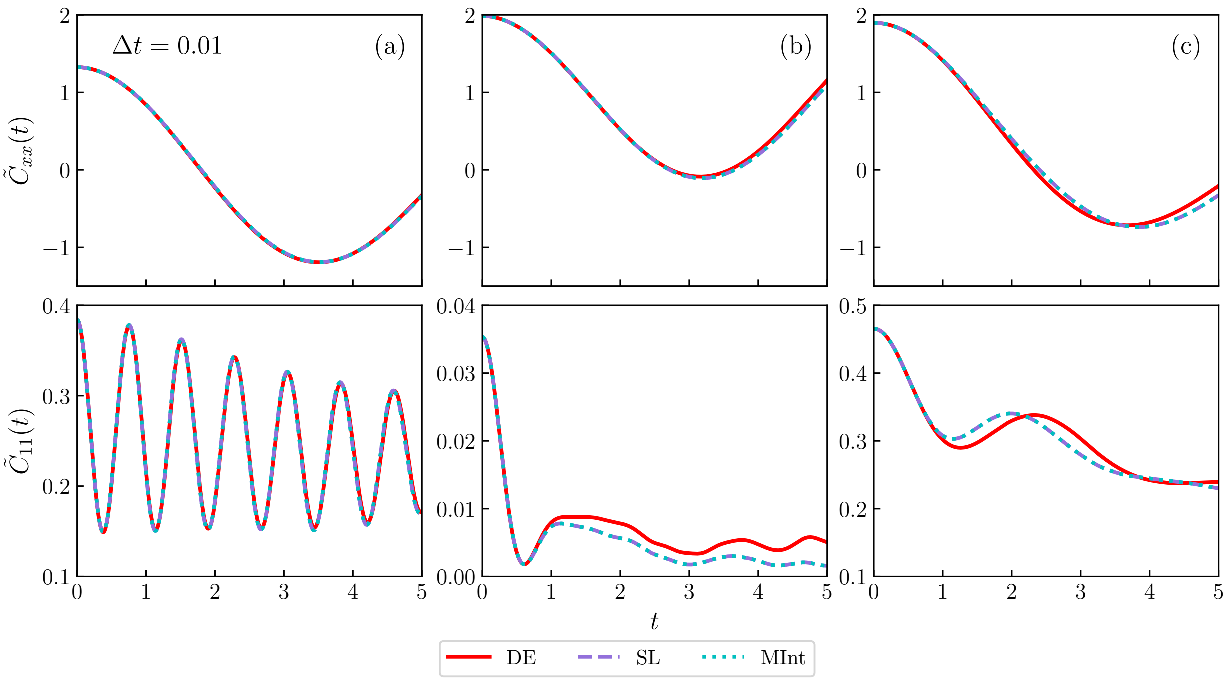

Figure S3: The nuclear position, $\tilde{C}_{xx}(t)$, and electronic population, $\tilde{C}_{11}(t)$, autocorrelation functions for various adiabatic couplings, (a) strong electronic coupling, (b) the inverted Marcus regime and (c) an intermediate regime, with the DE (red), the SL (purple) and MInt (cyan) algorithms using $\Delta t = 0.01$. The MInt and SL algorithms give identical results and the DE algorithm deviates from these results in the weaker coupling regimes.

Overall, the MInt and SL algorithms are very tolerant of a coarser timestep. Despite the MInt being slightly better using the coarse $\Delta t = 1.0$, this timestep does not capture the electronic oscillations well when there is strong electronic coupling so is unlikely to be used. Therefore, the MInt and SL algorithms are limited by the model used. The DE algorithm produces bad energy conservation for all timesteps tested and converges on the wrong long time values for the

electronic correlation functions. This indicates that the DE approximation leads to a systematic error and is not valid for the models tested here.

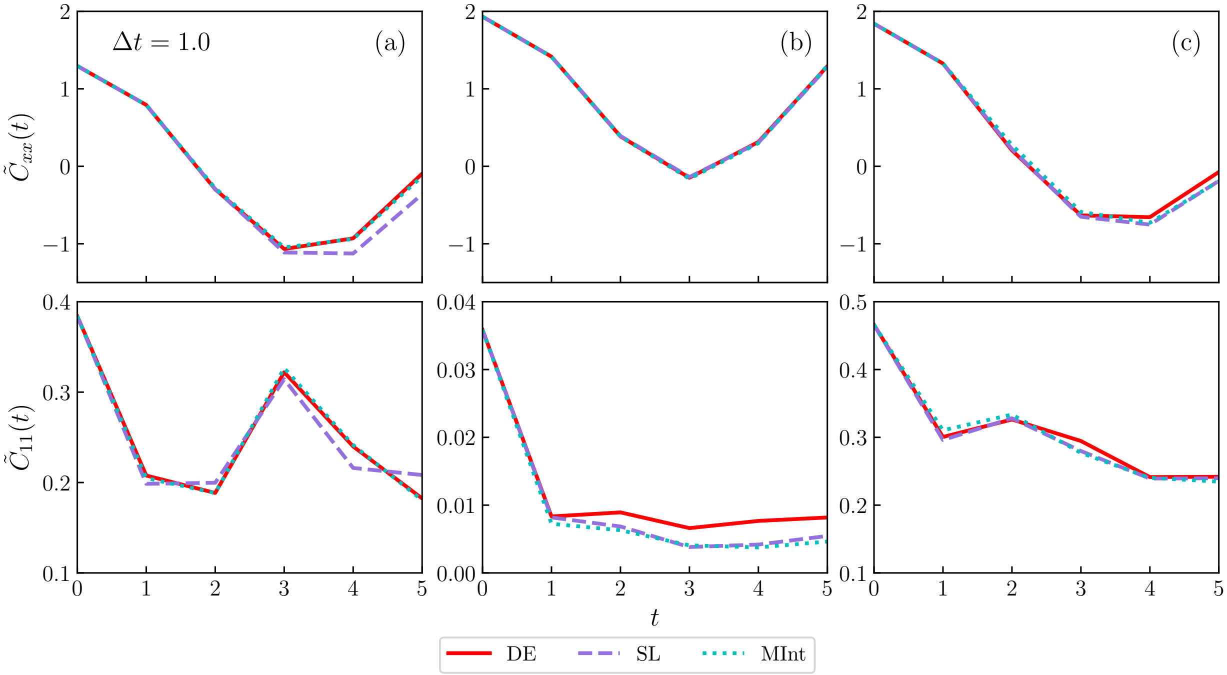

Figure S4: The nuclear position, $\tilde{C}_{xx}(t)$, and electronic population, $\tilde{C}_{11}(t)$, autocorrelation functions for various adiabatic couplings, (a) strong electronic coupling, (b) the inverted Marcus regime and (c) an intermediate regime, with the DE (red), the SL (purple) and MInt (cyan) algorithms using $\Delta t = 1.0$. We see that the MInt and SL results start to deviate from each other and the electronic correlation functions suffer from aliasing. The DE algorithm produces more similar results to the other two at this timestep.

Code Racing

In Table S1, we present code racing data for the three algorithms. We stress that this is only for comparison and we are not claiming to have the fastest implementation. The code is written in Python and was run on a Standard Compute Node on Myriad at UCL.

**Algorithm**	**With M /s**	**Without M /s**
SL	2355.64	334.37
MInt	2365.91	683.55
DE	3246.87	712.12

Table S1: Code racing results for the algorithms propagating 10000 trajectories for 5 time units using $\Delta t = 0.1$, with and without calculation of the monodromy matrix, $\mathbf{M}$. The SL is the fastest algorithm, followed by the MInt and DE. The DE algorithm is significantly slower with $\mathbf{M}$ compared to without.

We find that the MInt and SL algorithms are very similar when the monodromy matrix is calculated, but the SL is faster for the trajectory only. The DE algorithm is significantly slower than the other two when calculating the monodromy matrix but only slightly higher than the MInt without the monodromy matrix. This indicates that computing the monodromy matrix is significantly more costly than individual trajectories. For models with a very complex potential matrix, the cost is likely to even out for the algorithms as the bottleneck becomes computing the potential.

DE and MInt Original Form

Here, we compare the symplecticity and satisfaction of Liouville's theorem for the MInt and DE algorithms in their original propagation forms. This means the MInt flow map is,[2]

$$\Psi^{\mathrm{MInt}}_{H,\Delta t} := \Phi_{H_1,\frac{\Delta t}{2}} \circ \Phi_{H_2,\Delta t} \circ \Phi_{H_1,\frac{\Delta t}{2}}, \tag{1}$$

and the DE flow map is,[4]

$$\Psi^{\mathrm{DE}}_{H,\Delta t} := \Phi_{H_1,\frac{\Delta t}{2}} \circ \Psi_{\mathrm{DE},\Delta t} \circ \Phi_{H_1,\frac{\Delta t}{2}}, \tag{2}$$

where Φ refers to exact evolution, Ψ refers to approximate evolution which may or may not be comprised of exact sub-evolutions and $\Psi_{\mathrm{DE},\Delta t}$ refers to the approximate DE propagation of H_2.

Theoretically, as we determine the symplecticity and Liouville's theorem for H_1 and H_2 separately, the order of propagation should not change either property for a whole trajectory. We expect the DE algorithm to satisfy Liouville's theorem and be non-symplectic. Conversely, the MInt will be symplectic. Figure S5 confirms this, both algorithms satisfy Liouville's theorem and only the MInt is symplectic. The energy conservation is no different when using the original form, Figure S6, where we see large oscillations for the DE algorithm and much smaller oscillations for the MInt.

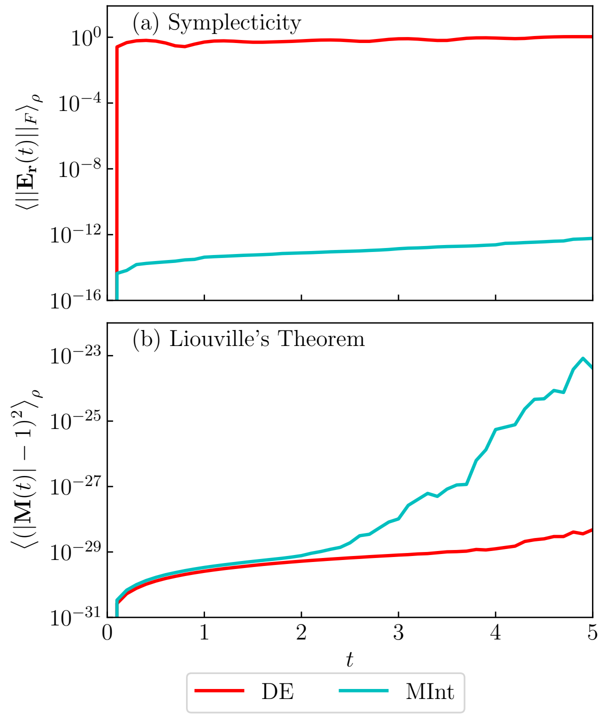

Figure S5: (a) The Frobenius Norm of the symplecticity error matrix and (b) the determinant criterion as a function of time using Model 1 and $\Delta t = 0.1$, averaged over a million trajectories using the MInt (cyan) and the DE (red) algorithms by propagating H_1, H_2 then H_1. In (a) the MInt algorithm is seen to be symplectic whereas the DE is not, whereas in (b) both algorithms satisfy Liouville's theorem.

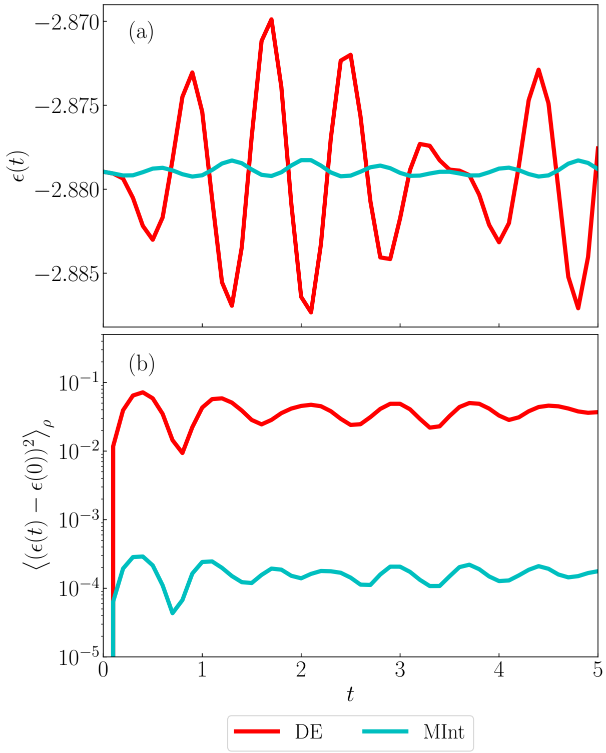

Figure S6: The energy conservation for Model 1 with (a) a single trajectory and (b) averaged using $\Delta t = 0.1$ with the MInt (cyan), and the DE (red) algorithms by propagating H_1, H_2 then H_1. The MInt and DE algorithms give the same energy conservation trends as for propagating H_2 first.

References

(1) Richardson, J. O.; Thoss, M. Communication: Nonadiabatic ring-polymer molecular dynamics. *J. Chem. Phys.* **2013**, *139*, 031102.

(2) Church, M. S.; Hele, T. J. H.; Ezra, G. S.; Ananth, N. Nonadiabatic semiclassical dynamics in the mixed quantum-classical initial value representation. *J. Chem. Phys.* **2018**, *148*, 102326.

(3) Richardson, J. O.; Meyer, P.; Pleinert, M.-O.; Thoss, M. An analysis of nonadiabatic ring-polymer molecular dynamics and its application to vibronic spectra. *Chem. Phys.* **2017**, *482*, 124–134.

(4) Kelly, A.; van Zon, R.; Schofield, J.; Kapral, R. Mapping quantum-classical Liouville equation: Projectors and trajectories. *J. Chem. Phys.* **2012**, *136*, 084101.
